# A Cross-Sectional Study on the Knowledge, Attitudes, and Oral Hygiene Practices of Secondary School Students in Al-Qunfudah District, Saudi Arabia

**DOI:** 10.7759/cureus.40337

**Published:** 2023-06-12

**Authors:** Safa H Alkalash, Athbah E Alfaqih, Ameera I Alkinani, Hanan M Alzahrani, Muneerah H Alrufaydi, Raghad S Alqarni, Manal H Alrufaydi

**Affiliations:** 1 Community Medicine and Healthcare, Umm Al-Qura University, Al-Qunfudah, SAU; 2 Family Medicine, Menoufia University, Shebin Elkom, EGY; 3 Medicine and Surgery, Umm Al-Qura University, Al-Qunfudah, SAU

**Keywords:** school students, saudi arabia, practices, oral hygiene, knowledge, attitudes

## Abstract

Background

Oral health is closely related to general health awareness and good oral hygiene practices, which makes it an important aspect of general health and well-being. The current study assessed the knowledge, attitudes, and practices of oral hygiene among secondary school students in Al-Qunfudah district, Saudi Arabia.

Methods

A convenient sample of 383 male and female secondary school students in the Al-Qunfudah district, Saudi Arabia, was included in this descriptive cross-sectional study. The research data were collected from the study sample through a self-administered online survey via WhatsApp and Telegram electronic applications. Finally, the collected data were coded and analyzed through the Statistical Package for the Social Sciences (SPSS) version 25.0 (SPSS Inc., Chicago, IL) and expressed in frequencies, percentages, mean, and standard deviation. The chi-squared and Fisher's exact tests were applied to assess the relationship between the participants' knowledge and practice scores of oral hygiene and their demographic characteristics. A P-value of less than 0.05 was regarded as significant.

Results

In this study, female students represented 70% of the sample, and most of them were Saudi (97.0%). Many of them identified the great impact of oral hygiene on overall health (89.2%), and more than two-thirds indicated that brushing their teeth regularly would prevent oral problems (89.2%). Nearly two-thirds of them perceived that dental problems would affect their school attendance; however, 59.1% ignored the important role of schools in maintaining students’ oral hygiene. The poor practice of dental care was observed among students, as 47.5% brush their teeth twice daily and a third replace their brushes every three months. Despite the fact that the majority of the study population had adequate knowledge (44.4%) and a positive attitude (78.6%) toward oral hygiene, only 39.9% could effectively apply it. The knowledge of oral hygiene was significantly better among females (P = 0.005), older students (P = 0.001), those at a higher academic level (P = 0.016), and students from Al-Qunfudah city (P = 0.007). Furthermore, older pupils, those with a higher academic level, and those from Al-Qunfudah city performed better in terms of dental hygiene than their peers (P-value was 0.001 for each).

Conclusion

Secondary school students generally have adequate awareness levels and positive attitudes toward oral hygiene, but they practice it with remarkably little consistency. Schools should emphasize their crucial roles in enhancing oral health among their students of different education levels through their curriculum and frequent monitoring of students. Persuasive and realistic oral health education initiatives are recommended for schoolchildren and their family members or caregivers, with a particular emphasis on males, younger children, and those living in rural areas.

## Introduction

Oral health is the condition of the mouth, teeth, and orofacial structures that allows people to perform essential functions like eating, breathing, and speaking, as well as psychosocial aspects like self-confidence, well-being, and the ability to socialize and work without pain, discomfort, or embarrassment [1]. On the other hand, disregard for oral health can result in torment and distress, which has a detrimental impact on people's lives and reduces their work output [2]. Nevertheless, as they are frequently acquired after conceptualizing oral health practices, knowledge about oral health practices alone does not guarantee that individuals' states of mind and actions would change as a result [2]. Good oral hygiene consists of the continuous implementation of two sets of behaviors: engaging in dental services (routine dental checkups, oral health promotion, and competently applied prevention strategies) and self-care habits (good oral hygiene, sugar restriction, and fluoride product application) [3]. To prevent oral health problems, it is recommended to brush their teeth twice a day with fluoride toothpaste, and adults should floss their teeth at least once a day and have a regular oral health checkup [2,3].

Despite significant advances in oral health, the global burden of oral health problems remains high [1,4]. This might be due to a lack of adoption of healthy oral practices, which are critical in controlling the most prevalent oral illnesses, such as dental caries and periodontal disease, both of which are considered behavioral disorders [5]. This oral health issue may be attributed to the rapid progression of dental disorders following alterations in lifestyle, such as the consumption of sugar-rich foods and beverages, the requirement for the addition of fluoride to water, and other social and environmental factors [6]. Oral health is a legitimate public health concern due to the high incidence and prevalence of oral diseases. In addition, oral illness medications are ranked as the fourth most expensive disease treatment in the majority of industrialized nations [7].

A large study by Peltzer and Pengpid in 2014 examined oral hygiene practices and related variables among undergraduates from various disciplines in more than 20 countries that have different socioeconomic levels (low-middle-high income) in order to better understand the awareness, perspectives, and skills of oral health among school students [8]. This study determined that the rates of oral hygiene and dental care were poor among college students from societies as diverse as those in Africa, Asia, and the Americas [8]. Although the respondents in a different study of high school students in Nigeria had high levels of knowledge, their attitudes and actual use of oral healthcare did not match those levels [9].

In Arab nations, several studies have been conducted to evaluate oral health knowledge, attitudes, and behaviors, particularly among school students. A Jordanian study revealed a higher level of understanding about dental caries than other conditions, while noncompliance with a regular examination by a dentist was widespread; furthermore, it explored that pain was the primary driver of dental appointments. Despite children's positive attitudes toward their dentists, they were scared of undergoing dental treatment [10]. In Egypt, a study was done among 408 primary school students to identify their oral hygiene practices and examine their dental status, and it was reported that 68% of students possessed decayed teeth as a result of inappropriate healthy practices such as proper tooth brushing, and it was reported that 68% of students possessed decayed teeth as a result of inappropriate healthy practices such as proper tooth brushing [11]. Previous studies in Saudi Arabia on this topic revealed variable degrees of knowledge and attitudes toward oral hygiene among school students, but most of them detected unsatisfactory practice [2,12]. Despite several previous studies concluding that secondary school students had poor knowledge and practice as regards oral hygiene [5,10,12], no previous studies regarding this important health-related issue were done in the Al-Qunfudah district; therefore, this study has been designed to assess the awareness, perspectives, and practices of secondary school students in Al-Qunfudah district, Saudi Arabia, regarding oral hygiene.

## Materials and methods

Study setting and subjects

A descriptive cross-sectional study was conducted among a convenient sample of 383 male and female secondary school students in Al-Qunfudah district which is the largest district of Al-Qunfudah governorate, Saudi Arabia. It is the administrative governorate center, located in the southwest of the Emirate of Makkah at 360 km. It has a population of 24,512, of which 8,570 are foreign immigrants. And the commercial core of the nearby communities is all government offices. The district of Al-Qunfudah is divided into eight communities or districts, and each community has a responsible elder who represents the people's interests before government institutions, and he is appointed by the governor. Al-Qunfudah district involves a total of 3,830 male and female secondary school students who are living in Al-Qunfudah city and the nearby villages [13].

Sample size calculation

The study sample size was estimated using the online sample size calculator software EPI-INFO [14], with a 5% accepted margin of error and a 95% confidence level. The overall number of secondary school students was 3,830, with a percentage of good knowledge of 59.1% [12]. The recommended sample size was 339 participants.

Data collection tool

For this study, data were obtained via a self-reported survey. After meticulously reviewing the literature and discussing it with the research group, the study's authors decided on the survey questions. It is divided into four components to evaluate the students' awareness, attitudes, and practices related to oral hygiene as well as their sociodemographic factors. The total items of this questionnaire are organized into four sections. The five sociodemographic questions that were addressed in the first segment were age, gender, nationality, place of residence, and degree of education. A total of 17 items were involved in the second section to assess their knowledge of oral hygiene, such as its effects on someone’s health, the number of permanent teeth, the frequency of changing toothbrushes, the definition of plaque, and the effect of bad breath on social activity. The third section of this survey was made up of 10 questions to assess the attitudes of participants toward oral and dental hygiene, the importance of regular tooth brushing, and the causes of dentist visits. The fourth and last portion had nine items that evaluated their practice to maintain oral health. After the survey had been established, it was subsequently tested before use in a pilot study to verify whether pupils would comprehend it and to determine response rates. The survey link was propagated among secondary school students by using the WhatsApp and Telegram applications of their educational groups and asking them to send the survey link to their peers in Al-Qunfudah secondary schools. We received the first 35 responses (representing about 10% of the sample size). There was no need to make any modifications to any of the survey's items since the survey's reliability had been evaluated with a Cronbach's alpha coefficient of 0.81.

Procedure for data collection

The study data were obtained during a two-month period (May and June 2022) through the distribution of the structured survey on WhatsApp and Telegram groups of the students and its dissemination to their classmates. The survey included a question regarding the place of residence, including whether respondents were from Al-Qunfudah city, Al-Qunfudah-related villages, or beyond the Al-Qunfudah district, in order to check that all data were collected from secondary school students in Al-Qunfudah district. It was intended to exclude those who were from areas other than the Al-Qunfudah district from the study's final results. Thirteen incomplete surveys were found after we sorted through the 396 submitted answers and removed them. Finally, 383 surveys in all were completed.

The study had been granted the HAPO-02-K-012-2022-02-977 ethical approval number from the College of Medicine's Medical Research and Ethics Committee at Umm Al-Qura University in Makkah, Saudi Arabia before it was put into action. Each student provided informed consent before the study's start. Data were gathered anonymously and kept safeguarded in secure electronic folders.

Data analysis

All statistical computations were performed using the Statistical Package for the Social Sciences (SPSS) version 25.0 (SPSS Inc., Chicago, IL). The entirety of the data was provided, coded, and double-checked for consistency. Each correct response received one point, while each incorrect response received zero points, to get the overall estimate of oral health knowledge, behavior, and practices. The knowledge score was divided into three categories: good (mean = 0.76-1.00), fair (mean = 0.51-0.75), and poor (mean = 0.00-0.50). Their practice score was classified into good practice (mean = 0.51-1.00) and poor practice (mean = 0.00-0.50), with good (positive) attitude being rated as having mean =0.51-1.00, while poor (negative) attitude was graded as having mean =0.00-0.50. To display descriptive statistics, frequencies, percentages, mean, and standard deviation were employed. The chi-squared test (X^2^) was used with qualitative data to show the relationship between the variables, and Fisher's exact test was used to examine whether there are any nonrandom relationships between two categorical variables. Statistical significance was established with a P-value of 0.05.

## Results

Regarding the sociodemographic characteristics of the secondary school students who were involved in the study, more than two-thirds of them were female (70.0%), Saudi nationality was predominant (96.9%), and nearly half belonged to the age group 15-16 years. The students from the nearby villages represented 53.8% of the sample (Table 1).

**Table 1:** Sociodemographic characteristics and clinical data of the studied subjects (n = 383)

Demographic characteristics	Frequency (N)	Percent
Gender		
Male	115	30.0
Female	268	70.0
Age group		
15-16 years	170	44.4
17-18 years	163	42.6
19-20 years	50	13.0
Education level		
First secondary	168	43.8
Second secondary	136	35.5
Third secondary	79	20.7
Nationality		
Saudi	371	96.9
Non-Saudi	12	3.1
Residence		
Inside Al-Qunfudah city	177	46.2
Al-Qunfudah-related villages	206	53.8

Most students reported that teeth are an important part of the human body (97.1%). Above two-thirds (89.2%) of them knew that brushing their teeth regularly would prevent oral problems, while 46.3% correctly knew that toothbrushes should be changed every three months, and the causes of dental plaque were well known to 44.9% of students. The students' knowledge regarding the recommended frequency of dental brushing and visiting a dentist was low (40.5% and 25.6%, respectively) (Table 2).

**Table 2:** Knowledge of studied subjects toward oral hygiene (n = 383)

Items	Frequency (N)	Percent
Teeth are an important part of human body		
Yes	372	97.1
No	4	1.0
Don't know	7	1.9
Oral health has an impact on overall human health		
Yes	342	89.2
No	13	3.4
Don't know	28	7.4
Brushing teeth regularly will prevent oral problems		
Yes	342	89.2
No	17	4.4
Don't know	24	6.4
Recommended frequency of teeth brushing every day		
Once	131	34.2
Twice	155	40.5
After each meal	55	14.3
Don’t know	42	11.0
Improper cleaning of the tongue leads to bad breath		
Yes	287	74.9
No	28	7.4
Don't know	68	17.7
Falling teeth will affect the way of speaking		
Yes	332	86.7
No	17	4.4
Not sure	34	8.9
Sweets/soft drinks cause mouth problems		
Yes	347	90.6
No	13	3.4
Don't know	23	5.9
Rinsing after eating, sweets, and drinks prevents mouth problems		
Yes	304	79.3
No	28	7.4
Don't know	51	13.3
Maintaining good oral hygiene prevents tooth decay		
Yes	368	96.1
No	13	3.4
Don't know	2	0.5
Tooth decay/tooth decay affects the appearance of the teeth		
Yes	362	94.6
No	13	3.4
Don't know	8	2.0
A dentist can help keep teeth healthy		
Yes	285	74.4
No	45	11.8
Don't know	53	13.8
Recommended frequency for anyone to attend dental clinic		
Every three months	30	7.8
Every six months	98	25.6
A year	95	24.8
When having dental pain	128	33.4
Don’t know	32	8.4
Total number of deciduous teeth		
20 Teeth	247	64.5
30 Teeth	109	28.5
32 Teeth	27	7.0
Total number of permanent teeth		
20 Teeth	30	7.8
30 Teeth	108	28.2
32 Teeth	245	64.0
Recommended frequency for changing the toothbrush		
Every month	181	47.3
Every three months	177	46.3
Every five months	25	6.4
Definition of dental plaque		
A layer of plaque builds up and becomes more solid	72	18.7
It consists of saliva, bacteria, and food, in addition to the acids	39	10.3
Formed by bacteria	100	26.1
All the above	172	44.9
The effect of dental plaque on the teeth		
Bad breath	26	6.8
Tooth decay and cavities	18	4.7
Gingivitis and periodontal disease	15	3.9
All the above	322	84.1

Secondary school students showed adequate levels of knowledge as regards the importance of teeth for individuals, the association between maintaining oral hygiene and whole body health, and the bad impact of dental decay on someone's appearance, with mean values of 0.97, 0.96, and 0.95, respectively, while their knowledge was poor about the recommended frequency of follow-up by a dentist and the frequency of changing toothbrushes, with mean values of 0.26 and 0.46, respectively (Table 3). 

**Table 3:** Participant’s level of knowledge about oral hygiene SD = standard deviation.

Statement		Mean	SD	Rank	Knowledge level
Teeth are an important part of human body	0.97		0.452	1	Good knowledge
Oral health has an impact on overall human health	0.89		0.465	5	Good knowledge
Brushing teeth regularly will prevent oral problems	0.89		0.385	6	Good knowledge
Recommended frequency of teeth brushing every day	0.40		0.265	16	Poor knowledge
Improper cleaning of the tongue leads to bad breath	0.75		0.495	11	Fair knowledge
Falling teeth will affect the way of speaking	0.87		0.501	7	Good knowledge
Sweets/soft drinks cause mouth problems	0.91		0.416	4	Good knowledge
Rinsing after eating, sweets, and drinks prevents mouth problems	0.79		0.484	9	Good knowledge
Maintaining good oral hygiene prevents tooth decay	0.96		0.415	2	Good knowledge
Tooth decay/tooth decay affects the appearance of the teeth	0.95		0.382	3	Good knowledge
A dentist can help keep teeth healthy	0.75		0.479	10	Fair knowledge
Recommended frequency for anyone to attend dental clinic	0.26		0.239	17	Poor knowledge
Total number of deciduous teeth	0.65		0.278	12	Fair knowledge
Total number of permanent teeth	0.64		0.378	13	Fair knowledge
Recommended frequency for changing the toothbrush	0.46		0.298	14	Poor knowledge
Definition of dental plaque	0.45		0.283	15	Poor knowledge
The effect of dental plaque on the teeth	0.84		0.361	8	Good knowledge

The majority considered brushing important for avoiding tooth decay (86.7%) and having fresh breath (67.9%). Additionally, 83% recognized that poor oral hygiene prevents them from smiling and laughing with friends. About two-thirds (67.2%) of the students thought that the only reason for visiting a dentist is dental pain, and more than half (59.1%) of them denied the role of schools in maintaining oral health (Table 4). 

**Table 4:** Attitude of school students toward oral hygiene (n = 383)

Items	Frequency (N)	Percent
Do you think brushing your teeth regularly twice daily can prevent dental caries?		
Yes	332	86.7
No	51	13.3
Do you think improper cleaning of the tongue results in bad breath?		
Yes	260	67.9
No	123	32.1
Do you think poor oral hygiene prevents you from smiling and laughing with friends?		
Yes	318	83.0
No	65	17.0
Do you think oral problems force you to miss school?		
Yes	234	61.1
No	149	38.9
Do you think maintaining healthy teeth is an individual's responsibility?		
Yes	351	91.6
No	32	8.4
Do you think school plays an important role in maintaining oral hygiene?		
Yes	157	40.9
No	226	59.1
Do you think regular visit to the dentist is necessary?		
Yes	342	89.2
No	41	10.8
Do you think dentist helps maintain oral health?		
Yes	351	91.6
No	32	8.4
Do you think your parents are the reason for your visit to the dentist?		
Yes	240	62.6
No	143	37.4
Do you think pain or discomfort is the only reason for you to visit a dentist?		
Yes	257	67.1
No	126	32.9

Positive attitudes were observed among participants toward the importance of teeth brushing on a regular basis, oral hygiene being one's responsibility, and the role of the dentist in dental care, with mean values of 0.87, 0.92, and 0.92, respectively, while negative attitudes were discovered among them regarding the role of school in maintaining oral health among students and the reason for visiting a dentist, with mean values of 0.41 and 0.33, respectively (Table 5).

**Table 5:** Attitude score of school students toward oral hygiene SD = standard deviation.

Items	Mean	SD	Attitude score
Do you think brushing your teeth regularly twice daily can prevent dental caries?	0.87	0.443	Positive attitude
Do you think improper cleaning of the tongue results in bad breath?	0.68	0.321	Positive attitude
Do you think poor oral hygiene prevents you from smiling and laughing with friends?	0.83	0.439	Positive attitude
Do you think oral problems force you to miss school?	0.61	0.282	Positive attitude
Do you think maintaining healthy teeth is an individual's responsibility?	0.92	0.354	Positive attitude
Do you think school plays an important role in maintaining oral hygiene?	0.41	0.281	Negative attitude
Do you think regular visit to the dentist is necessary?	0.89	0.213	Positive attitude
Do you think dentist helps maintain oral health?	0.92	0.283	Positive attitude
Do you think your parents are the reason for your visit to the dentist?	0.63	0.342	Positive attitude
Do you think pain or discomfort is the only reason for you to visit a dentist?	0.33	0.218	Negative attitude

About two-thirds of the study subjects (65.5%) did not brush their teeth regularly; 47.5% brushed their teeth twice daily, and the main reason they did not brush their teeth was their waking up late (64.0%). Two-thirds of the secondary school students (69.5%) did not clean their tongues regularly. About a third of the study subjects changed their toothbrushes every three months. The most commonly used teeth-cleansing agent (91.7%) was toothpaste, and about half of the participants used floss to clean their teeth (Table 6). 

**Table 6:** Practice of school students toward oral hygiene (n = 383)

Items	Frequency (N)	Percent
How many times do you brush your teeth per day?		
Occasionally	46	12.0
Once	155	40.5
Twice	182	47.5
Do you brush your teeth regularly?		
Yes	132	34.5
No	251	65.5
What is the reason for not brushing your teeth regularly?		
Laziness	130	33.9
Wake up late	245	64.0
Not useful	8	2.1
What do you use to brush your teeth regularly?		
Powder	12	3.1
Toothpaste	351	91.7
Toothpicks	20	5.2
Do your parents observe you during teeth brushing?		
Yes	56	14.6
Sometimes	157	41.0
No	170	44.4
How often do you change your brush?		
Per month	153	39.9
Every two months	66	17.2
Every three months	126	33.0
Every six months	38	9.9
Do you rinse your mouth after meals/sweets/soft drinks?		
Yes	298	77.8
No	85	22.2
Do you brush your tongue regularly?		
Yes	117	30.5
No	266	69.5
Do you use dental floss?		
Yes	194	50.7
No	189	49.3

Good students' practice was obvious as regards rinsing their teeth after sweets and soft drinks, using toothpaste, and flossing in dental cleaning with mean values of 0.78, 0.92, and 0.51, respectively, while they showed poor practice as regards cleaning their tongues, frequency, and adherence to tooth brushing with mean values of 0.31, 0.48, and 0.35 (Table 7).

**Table 7:** Practice level of school students toward oral hygiene SD = standard deviation.

Items	Mean	SD	Practice level
How many times do you brush your teeth per day?	0.48	0.231	Poor practice
Do you brush your teeth regularly?	0.35	0.283	Poor practice
What is the reason for not brushing your teeth regularly?	0.64	0.342	Good practice
What do you use to brush your teeth regularly?	0.92	0.465	Good practice
Do your parents observe you during teeth brushing?	041	0.223	Poor practice
How often do you change your brush?	0.33	0.289	Poor practice
Do you rinse your mouth after meals/sweets/soft drinks?	0.78	0.338	Good practice
Do you brush your tongue regularly?	0.31	0.228	Poor practice
Do you use dental floss?	0.51	0.453	Good practice

**Figure 1:**
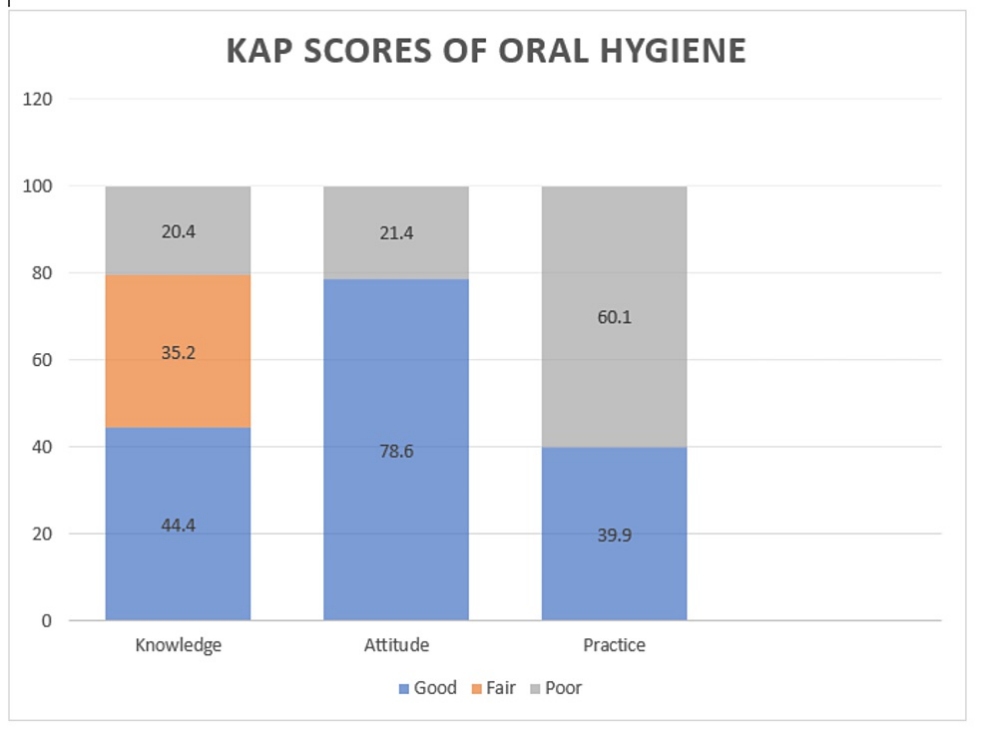
Knowledge, attitude, and practice (KAP) scores of oral hygiene among secondary school students

The overall participants' knowledge of oral hygiene was good at 44.4%, fair at 35.2%, and poor at 20.4%. Despite the fact that most of them (78.6%) had a positive attitude regarding oral hygiene, only 39.9% could practice it well (Figure 1). 

When the overall knowledge level of students about oral hygiene was compared to the sociodemographic characteristics of the participants, it was discovered that the knowledge levels of females, older students, students at a higher academic level, and residents of Al-Qunfudah city were higher than those of the other participants, with a significant difference found (P values = 0.005, 0.001, 0.016, and 0.007, respectively) (Table 8).

**Table 8:** Relationships between participants’ knowledge scores and sociodemographic data (n = 383) All values are presented as numbers (N) and percentages. Statistically significant (P-value < 0.05).

		Knowledge score			P-value
		Good knowledge, N (%)	Fair knowledge, N (%)	Poor knowledge, N (%)	
Gender	Male	45 (26.5)	54 (40.0)	16 (20.5)	0.005
	Female	125 (73.5)	81 (60.0)	62 (79.5)	
Age group	15-16 years	62 (36.5)	64 (47.4)	44 (56.4)	0.001
	17-18 years	74 (43.5)	61 (45.2)	28 (35.9)	
	19-20 years	34 (20.0)	10 (7.4)	6 (7.7)	
Educational level	First secondary	71 (41.8)	61 (45.2)	36 (46.2)	0.016
	Second secondary	67 (39.4)	36 (26.7)	33 (42.3)	
	Third secondary	32 (18.8)	38 (28.1)	9 (11.5)	
Nationality	Saudi	165 (97.1)	131 (97.0)	75 (96.2)	0.921
	Non-Saudi	5 (2.9)	4 (3.0)	3 (3.8)	
Residence	Inside Al-Qunfudah city	93 (54.7)	57 (42.2)	27 (34.6)	0.007
	Al-Qunfudah-related villages	77 (45.3)	78 (57.8)	51 (65.4)	

The current results showed that older students, those with a higher academic level, and those from Al-Qunfudah city could practice oral hygiene better than their peers (the P-value was less than 0.001 for each) (Table 9).

**Table 9:** Relationships between participants’ practice scores and sociodemographic data (n = 383) All values are presented as numbers (N) and percentages. Statistically significant (P-value < 0.05). *Fisher's exact test.

		Practice score		P-value
		Good practice, N (%)	Poor practice, N (%)	
Gender	Male	41 (26.8)	74 (32.2)	0.261
	Female	112 (73.2)	156 (67.8)	
Age group	15-16 years	66 (43.1)	104 (45.2)	<0.001
	17-18 years	54 (35.3)	109 (47.4)	
	19-20 years	33 (21.6)	17 (7.4)	
Educational level	First secondary	54 (35.3)	114 (49.6)	<0.001
	Second secondary	51 (33.3)	85 (36.9)	
	Third secondary	48 (31.4)	31 (13.5)	
Nationality	Saudi	145 (94.8)	226 (98.3)	0.072*
	Non-Saudi	8 (5.2)	4 (1.7)	
Residence	Inside Al-Qunfudah city	93 (60.8)	84 (36.5)	<0.001
	Al-Qunfudah-related villages	60 (39.2)	146 (63.5)	

## Discussion

The present investigation aimed to provide a comprehensive overview of oral health behaviors, knowledge, and attitudes among secondary school students in the Al-Qunfudah district, Saudi Arabia, to aid in the planning and evaluation of the oral health promotion program. There was a high awareness that teeth are an important part of the body and that oral health greatly affected whole-body health (97.1% and 89.2%). The response was high compared to what was previously reported by Al-Amiri et al., Al-Darwish, and Al-Qahtani et al. in similar populations in northern Jordan, Qatar, and Saudi Arabia [10,15,16]. Brushing teeth regularly maintains oral health, while irregular brushing is associated with toothache, as cited by 89.2% and 75.9% of students, which is the same as in Al-Darwish and Kannan et al.'s studies [15,17]. In contrast, only 52% of an African study’s respondents were aware that brushing protects against bleeding per gum; 30% of them agreed that sweets damage teeth, and 65% declared that cleaning teeth would prevent tooth decay [18]. This discrepancy may be due to the differences between the two study settings.

According to Choi et al., oral bad odor caused by oral factors can be effectively reduced by dental hygiene interventions like removing the unpleasant breath-causing tongue covering with a toothbrush or tongue scraper. As can be seen, removing tongue coating accurately and correctly is a crucial part of managing foul breath [19]. The association between oral cavity odor and cleaning the tongue was known by 74.9% of the students. More than 90% of the study subjects were aware that sweet and soft drinks are the leading causes of dental caries, and maintaining oral hygiene will prevent it as well as maintain their appearance. This result is much higher than that obtained by Al-Qahtani et al. in their study in Abha, Saudi Arabia, who found that 53.5% of school students were aware of the role of sweets and sugar in the occurrence of dental caries [16]. This great discrepancy in both studies’ findings may be related to the differences in the participant's characteristics, as their study recruited school students with ages ranging from 12 to 16 years, while our study subjects were between 15 and 20 years old. Two-thirds of the secondary school students knew accurately the correct number of deciduous and permanent teeth, which is much better than the knowledge of primary school students in a previously done study in a rural area (Thadig and Ad-Delam) of the Riyadh region, Saudi Arabia, where only 20.3% correctly knew that milky teeth are 20 in number and 28.2% mentioned that anyone should have 32 permanent teeth [20]. The key reason for the results’ variability between our study and theirs may be due to the differences in both studies’ subjects’ characteristics and settings: their sample was made up of primary school students in rural areas, while ours was made up of secondary school students, and about half of them were from urban areas.

Unfortunately, the student’s knowledge regarding the recommended frequency of dental brushing and visiting a dentist was low (40.5% and 33.4%, respectively). This outcome is in line with that from a Qatari study among school students, where 34.5% of the sample mentioned that teeth brushing should be done twice daily and only 25.4% of them said that the recommended dentist visit must be every six months [15]. Both points should be highlighted among school students to educate them about the recommended frequency of tooth brushing and dispel the myth that dentist visits are only related to dental pain. Plaque is a clinically defined yellow-grayish material that attaches tenaciously to intraoral hard surfaces and is made up of an extracellular matrix (ECM), a wide variety of bacterial species, and harmful microorganisms [21]. In general, dental plaque contains over 10^11^ germs per milligram, which can lead to a variety of dental diseases such as gingivitis, caries, and periodontitis [22]. Even though 84.1% of the secondary school students in the current study well identified the risky effect of dental plaque, only 44.9% understood the mechanism of its formation. This finding is better than that previously recorded by Shaheen et al. in their Saudi study, which found only 31.7% of students correctly identified the causes of dental plaque development [20].

Positive student attitudes were detected as regards the importance of regular brushing of teeth twice daily in preventing oral problems (86.7%), the association between poor oral hygiene and halitosis and smiling (67.9% and 83%), the significant role of dentists, and adherence to their regular visits (91.6% and 89.2% of the study subjects, respectively). These findings are supported by several primary studies that detected positive attitudes among different school students [2,3]. These results are outstanding, and their positive attitudes may push them to improve their practice of oral hygiene. Most students (91.7%) believed that preserving oral health was mostly their responsibility, which corresponds to the perception of 99.4% of Indian students in a prior survey [23]. In this study, more than half of its sample denied the role of schools in maintaining students’ oral hygiene; this finding is supported by data obtained previously in Saudi and Qatari studies, which found that only 22.3% and 5.1% of the students knew about oral hygiene from their schoolteachers, while the main source of their knowledge came from their parents [5,15]. The school's role in educating students about oral hygiene should be enforced and maintained. Around two-thirds of students perceived that their parents were responsible for their visits to the dentist, which is contrary to what was detected by Al-Omiri et al. in Jordan, who found minimal guidance from parents concerning the children's dental hygiene practices [10]. This reflects the Saudi parents’ awareness and their interest in their children’s health. The only reason to attend dental clinics is dental pain or discomfort, as cited by 61% of secondary school students, which goes in line with the findings of Al-Omiri et al. and Kannan et al., who found that toothache was the major driving factor for dental visits [10,17].

Nearly half of the secondary school students in this study were used to brushing their teeth twice daily, but unfortunately, 65.5% did not practice this every day. This result matches that of an Egyptian study, where 65.7% of primary school children brush their teeth twice daily, while in Saudi Arabia, 48.3% of children clean their teeth whenever it is convenient rather than first thing in the morning or just before going to bed, and furthermore, just 39.1% of Indian schoolchildren brush their teeth twice daily [11,17,24]. This finding indicates the urgent need to reinforce children's regular brushing of their teeth twice daily. A third of secondary school students are accustomed to replacing their toothbrushes once every three months, which is the same as an Indian study that found around 43.4% of children changed their toothbrush once every six months and 30.9% of children changed their toothbrush every three months [23]. The oral surfaces are colonized by over 500 bacterial species, and the tongue has the largest bacterial load of any oral tissue and makes the greatest contribution to the bacteria found in the oral cavity. These bacteria contribute to plaque formation, so reducing the load of bacteria on the tongue may help reduce the rate of plaque formation on the teeth [25,26]. Cleaning the tongue is one of the important oral health practices that is ignored by 69.5% of the study subjects. This finding is in contrast with that reported by Azodo et al. in their study among students at the Federal School of Dental Therapy and Technology, Nigeria, which revealed that 81.8% of their study sample used a toothbrush to clean their tongues regularly [27]. This great difference between our and their results may be due to the characteristics of both study subjects, which are dental technology students who learn about oral health. It is very beneficial to clarify for the public the association between tongue hygiene and the prevention of dental plaque.

The most common procedure for cleaning teeth is brushing with toothpaste (91.6%), while dental flossing was done by 50.7% of the study subjects, which reflects that using toothpaste was preferred over other procedures. Similar findings were reported in Jordan, Egypt, Saudi Arabia, and India, where 83%, 99.6%, 72.7%, and 100% of the participating children used a toothbrush and toothpaste, while the same studies found that dental floss was rarely used among their sample [10,11,17,24]. Nearly half of the students (44.4%) who completed this survey did not have guidance from parents when cleaning their teeth, which is the same as the result of another previous Saudi study (48.3%) [17]. This outcome may be because older children make their parents leave them to fend for themselves, and this highlights the need to educate parents about their important role in monitoring their children while they brush their teeth from time to time and guiding them to improve their practice of oral hygiene.

Similar to the findings of earlier studies [12,15], female, older, and urban students performed better than their counterparts in terms of knowledge and practice of oral hygiene. This outcome was anticipated since older children generally have more information than younger kids, and because female teenagers often worry about their tooth health as part of their body image, this makes them keener to know appropriate oral hygiene and become more skilled in practicing it. Additionally, those who live in cities have more opportunities to attend health education classes about health-related issues like oral hygiene.

Limitations

This study has a number of limitations, the first of which is the sample's unequal stratification of students by gender, as most of the study respondents were female students. This pitfall could be eliminated by allocating both genders proportionally based on the total number of students from both genders and incorporating it as an inclusion criterion for sample selection, in addition to persuading male students to participate in the study through increased communication with them about its purpose and importance for them and others in their community. The second issue was selection bias, which was induced by the convenience (nonrandom) sample approach used; hence, employing one of the random sampling procedures might correct sample-related bias. Finally, the virtual nature of the data collection may compromise its validity, so it is preferable to perform a face-to-face interview with the study subjects to confirm the credibility of the acquired data. Despite the limitations noted above, this study is an initiative for more research in this isolated region, and it seems to be a spotlight to highlight the poor oral hygiene practice among adolescents in such a remote area.

## Conclusions

According to the findings of this survey, awareness and attitude among secondary school students as regards oral hygiene are adequate, while poor practice is evident among most of them. Female, older, and urban students had better knowledge and practice than their peers. It is necessary to emphasize the importance of oral health education for school students, ensuring that school-based oral health promotion programs are implemented continuously. Prior assessments of oral health education programs indicate that these programs can only provide short-term improvements in oral health behavior and status; hence, it is critical to repeat these programs and apply positive reinforcement to pupils. The oral health education program must be inspiring, lively, practical, and closely related to the learning capabilities created by the student at each educational level. Dental health education should be integrated into the current curriculum. Additionally, public health education campaigns about oral hygiene are recommended, with a focus on males and young children and their caregivers, especially those from rural communities. Furthermore, we recommend regular dental examinations every six months as an item of the school health program through collaboration between the ministries of health and education. In the future, more surveys should be conducted on a larger scale among school students and their parents or caregivers, and the data obtained should be used to develop better dental health programs.
